# Transcriptome Analyses Reveal Candidate Genes Potentially Involved in Al Stress Response in Alfalfa

**DOI:** 10.3389/fpls.2017.00026

**Published:** 2017-02-02

**Authors:** Wenxian Liu, Conghui Xiong, Longfeng Yan, Zhengshe Zhang, Lichao Ma, Yanrong Wang, Yajie Liu, Zhipeng Liu

**Affiliations:** ^1^State Key Laboratory of Grassland Agro-ecosystems, College of Pastoral Agriculture Science and Technology, Lanzhou UniversityLanzhou, China; ^2^Key Laboratory of Mineral Resources in Western China (Gansu Province), School of Earth Sciences, Lanzhou UniversityLanzhou, China; ^3^Ministry of Education Key Laboratory of Cell Activities and Stress Adaptations, School of Life Sciences, Lanzhou UniversityLanzhou, China

**Keywords:** alfalfa, aluminum stress, transcriptome, differentially expressed genes, RNA-Seq, internal detoxification mechanism

## Abstract

Alfalfa is the most extensively cultivated forage legume, yet most alfalfa cultivars are not aluminum tolerant, and the molecular mechanisms underlying alfalfa responses to Al stress are largely unknown. In this study, we aimed to understand how alfalfa responds to Al stress by identifying and analyzing Al-stress-responsive genes in alfalfa roots at the whole-genome scale. The transcriptome changes in alfalfa roots under Al stress for 4, 8, or 24 h were analyzed using Illumina high-throughput sequencing platforms. A total of 2464 differentially expressed genes (DEGs) were identified, and most were up-regulated at early (4 h) and/or late (24 h) Al exposure time points rather than at the middle exposure time point (8 h). Metabolic pathway enrichment analysis demonstrated that the DEGs involved in ribosome, protein biosynthesis, and process, the citrate cycle, membrane transport, and hormonal regulation were preferentially enriched and regulated. Biosynthesis inhibition and signal transduction downstream of auxin- and ethylene-mediated signals occur during alfalfa responses to root growth inhibition. The internal Al detoxification mechanisms play important roles in alfalfa roots under Al stress. These findings provide valuable information for identifying and characterizing important components in the Al signaling network in alfalfa and enhance understanding of the molecular mechanisms underlying alfalfa responses to Al stress.

## Introduction

Aluminum (Al) is a light metal and the third most abundant element in the earth's crust (Ma, 2005). When the pH in the soil is lower than 5.0, Al is dissolved from the harmless form (oxide or aluminosilicate form) into the soil solution, mostly in the form of Al^3+^, and results in phytotoxic to most herbaceous plants even at low concentrations. In the tropical and subtropical regions, Al toxicity has been considered as the major factor limiting crop production in acidic soils, which account for 40% of the world's arable land (Kochian et al., 2005). Traditionally, the application of large quantities of lime always used to alleviate the soil Al toxicity and then sustain the crop production. However, this practice is expensive and being unsustainable and not environmentally friendly. Thus, understanding the nature of Al tolerance mechanisms in plants and then developing cultivars with improved tolerance to acidic soil stress is an appealing approach to addressing this issue.

Up to data, they are two main Al tolerance mechanisms in plants, namely, the exclusion mechanism and tolerance mechanism, have been proposed. The secretion of certain organic acids, such as citrate, oxalate and malate, are induced by Al is one of the best proved characterization in exclusion mechanism in plants. In Al stress conditions, organic acids able to form strong complexes with Al^3+^ and then preventing the binding of Al to cellular components to alleviate Al toxicity (Ma et al., 2001; Ma, 2005). Many agriculturally important Al-tolerant plant species, such as wheat (*Triticum aestivum*) (Delhaize et al., 1993), snapbean (*Phaseolus vulgaris*) (Miyasaka et al., 1991), maize (*Zea mays*) (Pellet et al., 1995), *Cassia tora* (Ma et al., 1997a), soybean (*Glycine max*) (Yang et al., 2000), buckwheat (*Fagopyrum esculentum*) (Ma et al., 1997b), taro (*Colocasia esculenta*) (Ma, 1998), and rye (*Secale cereal*) (Li et al., 2000) release one or two of these three organic acids in response to Al stress. Moreover, the importance of high level of ascorbic acid to Al tolerance also has been indicated in transgenic tobacco (*Nicotiana tabacum*) (Yin et al., 2010). Through genetic and molecular analyses, various functional genes have been identified and confirmed as important components in Al tolerance. *ALMT1* is an Al-activated malate transporter gene identified from wheat. Overexpression of this gene in barley confers an Al-activated efflux of malate and results in the Al tolerance both in hydroponic culture and acid soil (Delhaize et al., 2004). When a *Pseudomonas aeruginosa* derived citrate synthase (CS) gene transformed into tobacco genome, higher citrate synthase activity, citrate efflux and greater Al resistance are observed in transgenic lines (de la Fuente et al., 1997). *AtALS3* (aluminum-sensitive 3) encodes a phloem-localized ABC transporter-like protein, which is required for Al resistance/tolerance in *Arabidopsis* by redistributing accumulated Al^3+^ away from sensitive tissues, such as root, and thus reducing the toxic effects of Al (Larsen et al., 2005). In rice (*Oryza sativa*), the disruption of *OsASR5* gene resulted in hypersensitivity to Al toxicity, and which may function as a transcription factor to protect rice cells from Al toxicity by regulating the expression of various genes (Arenhart et al., 2013). Recently, Yang et al. (2014) has shown auxin is responsible for the Al-induced inhibition of root growth and acts as the downstream of ethylene-regulated TAA1 expression in the root-apex transition zone.

Considering its complexity, it is essential to interpret the functional elements and molecular constituents involved in Al tolerance mechanisms on a whole-genome level in plants. Using microarray technology, a large number of Al-responsive genes in many plant species including *Arabidopsis* (Kumari et al., 2008), soybean (Duressa et al., 2010, 2011; You et al., 2011), wheat (Houde and Diallo, 2008), and *Medicago truncatula* (Chandran et al., 2008) have been identified. In addition, the recently developed high-throughput sequencing (RNA-Seq) has clear advantages over microarray methods and has been considered as the ideal option to discover new genes and estimate transcript abundance at genome-wide scale, especially useful for species without genome sequence (Trapnell et al., 2012; Zeng et al., 2015). Based on RNA-Seq platforms, genome-scale transcriptome analyses have been used to identify Al-stress-responsive genes in rice (Arenhart et al., 2014), buckwheat (*Fagopyrum tataricum*) (Yokosho et al., 2014; Zhu et al., 2015), maize (Mattiello et al., 2014), *Anthoxanthum odoratum* (Gould et al., 2015), and *Medicago truncatula* (Chen et al., 2012). These Al-stress-responsive genes identified by RNA-Seq are involved in many physiological and metabolic processes, such as protection against cell wall toxicity and oxidative stress, organic acid metabolism, and exudation, Al transportation, and hormone signal transduction.

Alfalfa (*Medicago sativa* L.) is the most extensively cultivated forage legume and plays vital ecological and economic roles in agricultural systems around the world (Liu et al., 2016). However, alfalfa is very sensitive to soil acidity, which greatly limits its productivity and persistence performance (Khu et al., 2013). Thus, a better understanding of the molecular mechanisms involved in alfalfa responses to Al stress would be critical for Al-tolerant alfalfa breeding programs. Previous studies have shown that overexpression of endogenous malate dehydrogenase or bacterial CS result in enhanced organic acid synthesis, Al secretion and Al resistance (Tesfaye et al., 2001; Barone et al., 2008). In addition to these transgenic studies, a proteomic analysis of alfalfa after Al treatment has revealed that leaf proteins responding to the Al stress are mainly involved in energy metabolism and antioxidant/reactive oxygen species (ROS) detoxification pathways (Rahman et al., 2014). Given the relatively low-throughput characteristics of proteomics-based approaches and the complex nature of the Al stress and resistance mechanisms, it is necessary to identify and functionally characterize Al-responsive genes in alfalfa on a genome-wide scale. Currently, to our knowledge, the genome-wide transcriptomic analysis of the Al-responsive genes in alfalfa has not been reported, especially within the root tips, where the primary site for Al detoxification and accumulation. Thus, in the present study, we carried out the first global transcriptome analysis of the alfalfa root tips during Al treatments using the Illumina RNA-Seq method. The results obtained in this study will extend the knowledge of the genetic basis of alfalfa response to Al stress at the transcriptional level.

## Materials and methods

### Plant materials, growing conditions, and treatments

Alfalfa seeds (cultivar Zhongmu No. 1) were surface sterilized in 1.0% (*v*/*v*) sodium hypochlorite for 5 min, rinsed 5 times with distilled water, and then germinated in deionized water-moistened standard germination paper for 3 days at 25°C in the dark. Seedlings with uniform tap root lengths were selected and hydroponically grown in an aerated nutrient solution as described by Chen et al. (2012). The pH of the culture solution was adjusted and maintained at 4.5 for the duration of the experiment. After 7 days of culture in a growth chamber at 25°C with a photoperiod of 16 h light/8 h dark, the seedlings were incubated in a 0.5 mM CaCl_2_ solution overnight. Then, 120 seedlings were separated averagely into four groups, which included three Al-treatment time point groups [4 (A4), 8 (A8), and 24 (A24) h] in a 0.5 mM CaCl_2_ solution containing 10 mM AlCl_3_ (pH 4.5) and one control (C) group, which was cultivated for 24 h in a 0.5 mM CaCl_2_ solution (pH 4.5). To reduce the circadian rhythm effects, the seedlings of A24 and C group were treated at the same time, and harvested after 24 h. For A8 and A4, their seedlings began to be treated 16 and 20 h after the treat time of A24, respectively, and harvested at the same time as A24 and C. Root tips (approximately 1.5 cm in length) were collected and immediately frozen in liquid nitrogen and stored at −80°C.

### cDAN library construction and sequencing

The RNA extraction, quality, and quantity measurement were performed according to previously described methods (Liu et al., 2016). After treated with DNase I (TaKaRa, Dalian, China), the total RNA was isolated with Sera-mag Magnetic Oligo (dT) Beads (Illumina) and a total of 6 μg derived mRNAs were fragmented and used for double-stranded cDNA library construction with random hexamer (N6) primers (Illumina). The cDNA library was sequenced with a read length of 100 nt (paired-end) using the Illumina HiSeq2000 System at the Beijing Genomics Institute (BGI)-Shenzhen, China (http://www.genomics.cn/index).

### Sequence filtering, assembly and annotation

Clean reads were obtained by filtering the adapter sequences and removing low-quality sequences with ambiguous “N” bases and reads with low *Q*-value (≤ 10) percentages more than 20% using the FASTX toolkit (http://hannonlab.cshl.edu/fastx_toolkit/). Trinity program was used for the *De novo* transcriptome assembly of quality reads into unigenes (Grabherr et al., 2011). To annotate the assembled unigenes, BLAST searches (*E* < 10^−5^) between unigenes and various databases like NCBI non-redundant nucleotide sequences (Nt), NCBI non-redundant protein sequences (Nr), Swiss-Prot, Clusters of Orthologous Groups of proteins (COG), Gene Ontology (GO), and Kyoto Encyclopedia of Genes and Genomes (KEGG) databases were performed, and the unigene sequence orientation was determined by the best results against the protein databases with a priority order of Nr > Swiss-Prot > KEGG > COG. If there is no significant hit against above databases, the coding regions and the sequence orientation were further predicted by ESTScan software (Iseli et al., 1999). Based on Nr annotation, GO annotation regarding biological process, molecular function, and cellular component were obtained using Blast2GO software (Conesa et al., 2005), and the GO functional classification was classified by WEGO (Ye et al., 2006).

### Differentially expressed gene analysis

SOAPaligner (v2.21) was used to map the reads to the assembled sequences and calculate the counts for each unigene (Li et al., 2009). Unigene expression level was determined using the Fragments Per Kilobase per Million Fragments mapped (FPKM) method (Mortazavi et al., 2008). The transcript fold change was calculated by the formula of log_2_ (FPKM-treatment/FPKM-control) using an MA-plot-based method with the random sampling model in the R package DEGseq (Wang et al., 2010). Then, differentially expressed genes (DEGs) were restricted with the absolute value of fold change ≥ 4 and False Discovery Rate (FDR) ≤ 0.0001 as the threshod by performing pairwise comparisons of Al treated samples with control sample. To examine the expression profile of DEGs, the expression data υ (C, A4, A8, and A24) were normalized to 0, log_2_ (υ4/υc), log_2_ (υ8/υc), and log_2_ (υ24/υc) and clustered using Short Time-series Expression Miner (STEM) software with a *p* ≤ 0.05 (Ernst and Bar-Joseph, 2006). The GO and KEGG pathway enrichment analysis for DEGs were performed using agriGO (http://bioinfo.cau.edu.cn/agriGO/) (Du et al., 2010) and KOBAS 2.0 (http://kobas.cbi.pku.edu.cn/) (Xie et al., 2011), respectively.

### Quantitative real-time PCR (qRT-PCR) analysis

Total RNA of C and A4 sample used for the RNA-Seq analysis were also used for qRT-PCR validation. The single-strand cDNAs used for qRT-PCR were synthesized from 2.5 μg of total RNA with MMLV reverse transcriptase (TaKaRa, Dalian, China). The qRT-PCR was performed using SYBR Premix Ex Taq II Kit (TaKaRa, Dalian, China) on a 7500 Fast Real-time PCR system (Applied Biosystems, USA). Fifteen genes involved in ribosome, TCA cycle, and molecule transport were selected for the qRT-PCR assays. Gene-specific primers were designed using Primer Express software (Applied Biosystems) and are shown in Table S1. Three technical replicates were carried out for each sample, and the relative expression levels were normalized to the expression of the *Medicago actin* gene (AA660796) and calculated using the 2^−ΔΔCT^ method.

## Results

### Transcriptome sequencing, assembly and annotation

In order to gain a general overview of the gene expression profiles of alfalfa roots under Al stress, four cDNA libraries representing one control (C, without Al stress) and three treatments at different time points (A4, A8, and A24) were designed for high-throughput RNA-Seq, and a total of 221,271,740 raw reads were ultimately obtained (Table 1). After removing the adaptor sequences, the ambiguous nucleotides and low-quality sequences, a total of 210,270,746 high quality clean reads remained, constituting over 15.9 GBase, with each library more than 4.0 GBase (Table 1). With the Trinity assembly software, a total of 185,454 contigs (≥ 200 bp) were obtained, and the contig sizes ranged from 200 to 10,138 bp, with a mean size of 612 bp. There were 29,626 (19.97%) contigs longer than 1000 bp. The more detail of the quality of the assembly transcripts is shown in Figure S1.

**Table 1:** **Summary of the sequence data analysis**.

**Sample**	**Total raw reads**	**Total clean reads**	**Total clean nucleotides (nt)**	**Q20 (%)**	**Unigenes**
C	54,642,438	54,642,438	4.9G	98.63	62,906
A4	45,729,878	45,729,878	4.1G	98.61	62,166
A8	61,211,992	54,849,606	4.9G	98.53	61.209
A24	59,687,432	55,048,824	5.0G	98.55	61,862
Summary	221,271,740	210,270,746	15.9G		75,903

**Figure 1:**
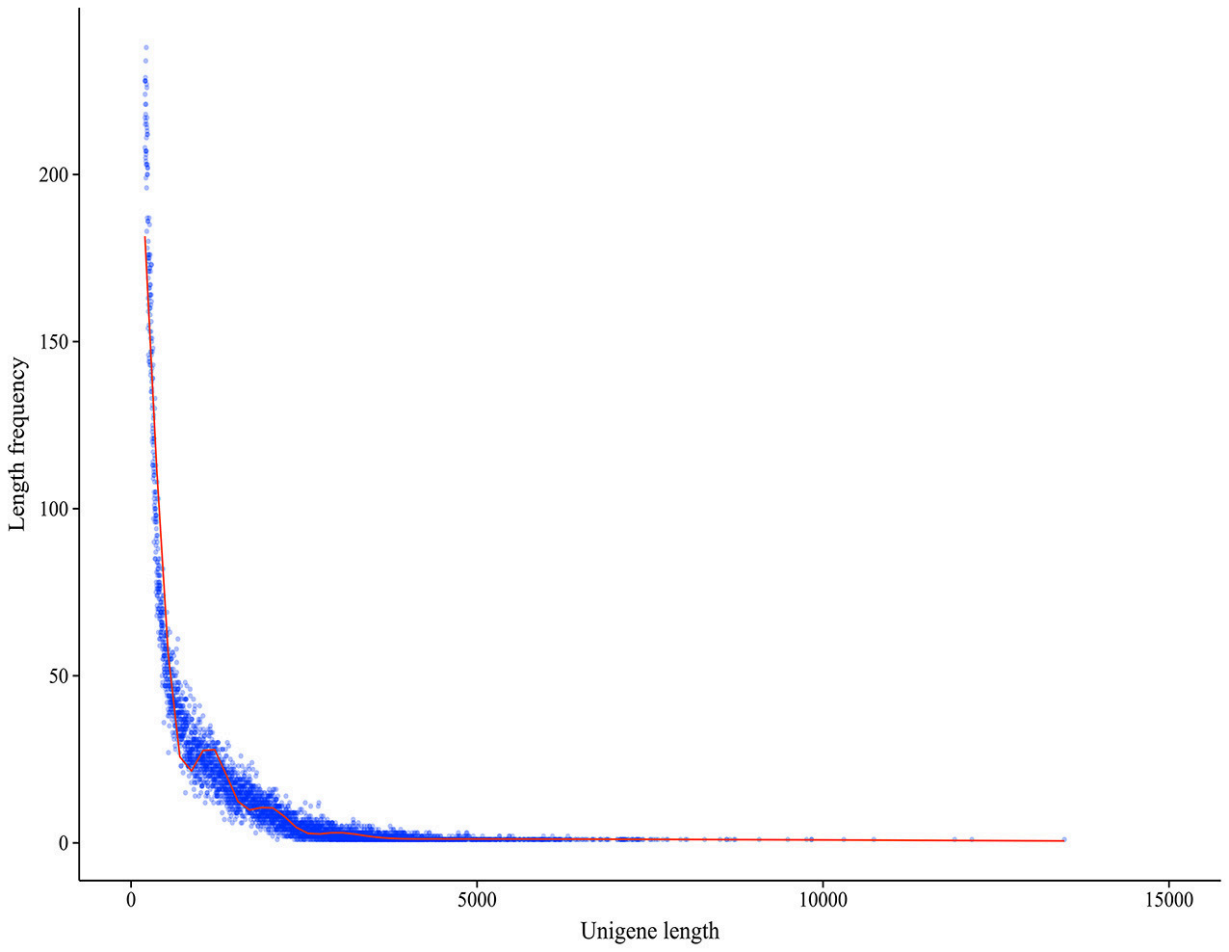
**Length distribution of the assembled unigenes**.

A total of 75,903 all-unigenes were assembled from the total contig assembly, and 62,906, 62,166, 61,209, and 61,862 unigenes were identified for the C, A4, A8, and A24 groups, respectively (Table 1). The length of these 75,903 all-unigenes ranged from 200 to 13,488 bp, with an N50 length of 968 bp (Figure 1). The relationships among the unigenes from the three treatments and the control are shown in Figure S2. For the functional annotation of all the unigenes, BLAST searches (*E* ≤ 10^−5^) against six public databases revealed that the number of the unigenes with significant similarity to the sequences in these databases ranged from 16,131 (21.25%, COG) to 54,075 (71.24%, Nt). Among the all-unigenes, 65,055 (85.71%) and 11,494 (15.14%) unigenes were annotated in at least one database and in all six databases, respectively (Table 2).

**Table 2:** **BLAST analysis of the non-redundant unigenes against public databases**.

**Annotated database**	**Number of unigenes**	**Percentage (%)**
Annotated in Nr	48,339	63.69
Annotated in Nt	54,075	71.24
Annotated in SwissProt	29,385	38.71
Annotated in GO	35,807	47.17
Annotated in KEGG	25,386	33.45
Annotated in COG	16,131	21.25
Annotated in all databases	11,494	15.14
Annotated in at least one database	65,055	85.71
Total unigenes	75,903	100

Among the 75,903 all-unigenes, nearly half of them (35,807) were assigned to 5288 GO annotations, and grouped into three main categories (Table S2). In the biological process (BP), unigenes were highly represented in “cellular process” (21,761), “metabolic process” (21,195), and “response to stimulus” (10,029). Within the cellular component (CC), “cell” and “cell part” (24,047 for both) were the most abundant groups, followed by “organelle” (17,692). For the molecular function (MF), the top 3 highly represented terms are “binding” (19,564), “catalytic activity” (18,332), and “transporter activity” (2128).

All of the unigenes were further assigned to the COG and KEGG pathway databases. A total of 16,131 unigenes were assigned to 25 COG functional classes (Figure S3). The largest group was “General functional prediction only,” followed by “Replication and repair,” “Transcription,” “Translation,” and “Post-translational modification, protein turnover, chaperon.” In addition, 25,386 all-unigenes were annotated in 276 individual KEGG pathways, with “Signal transduction” (5811, 4.39%) most highly represented, followed by “Translation” (2672, 6.62%), “Carbohydrate metabolism” (2638, 6.53%), and “Endocrine system” (2264, 5.61%) (Figure S4).

### DEGs in response to Al stress

**Figure 2:**
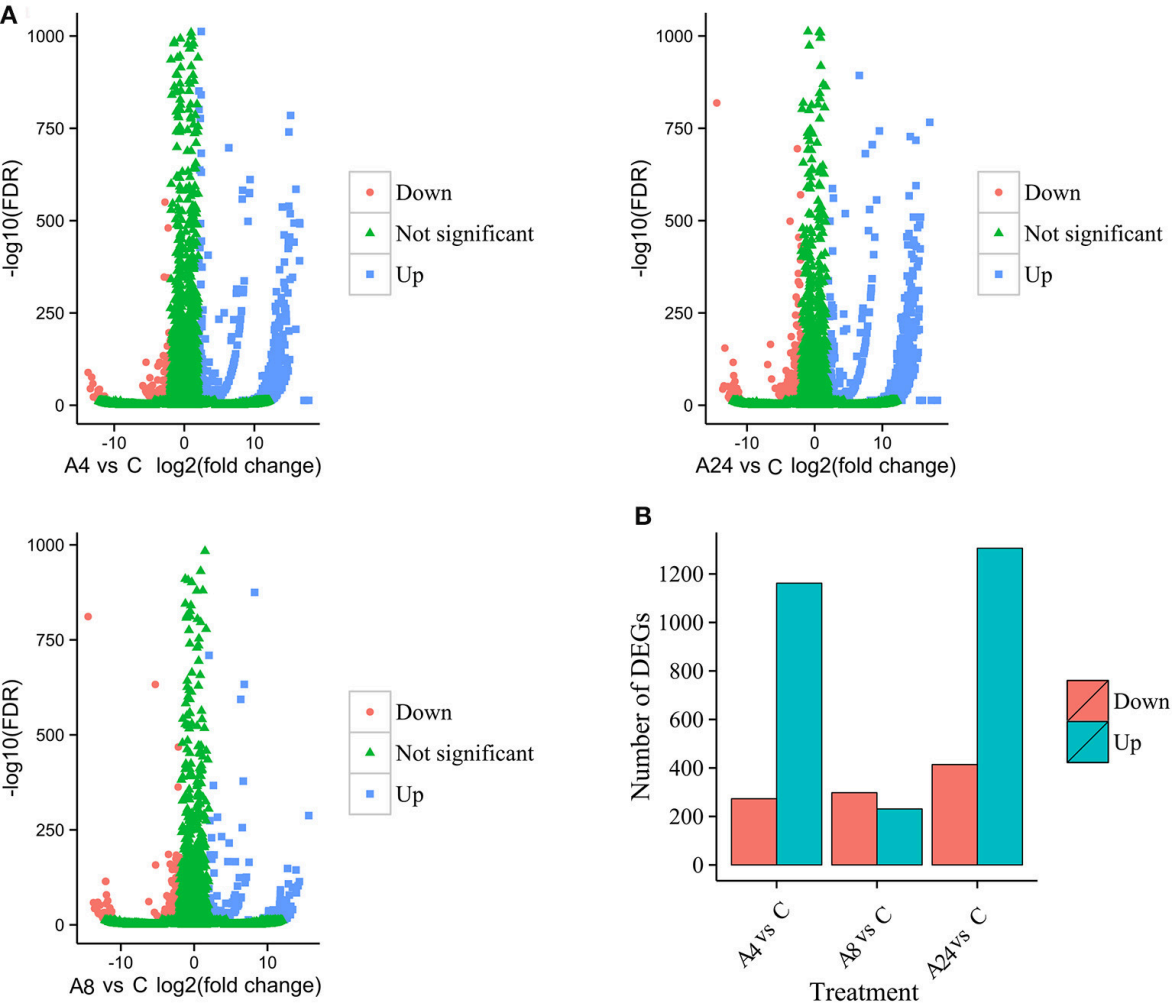
**Identification of the DEGs in response to Al stress. (A)** Volcano plots display log_2_ converted fold changes and FDR values. **(B)** The number of up- and down-regulated DEGs at each treatment time point compared with the control.

**Figure 3:**
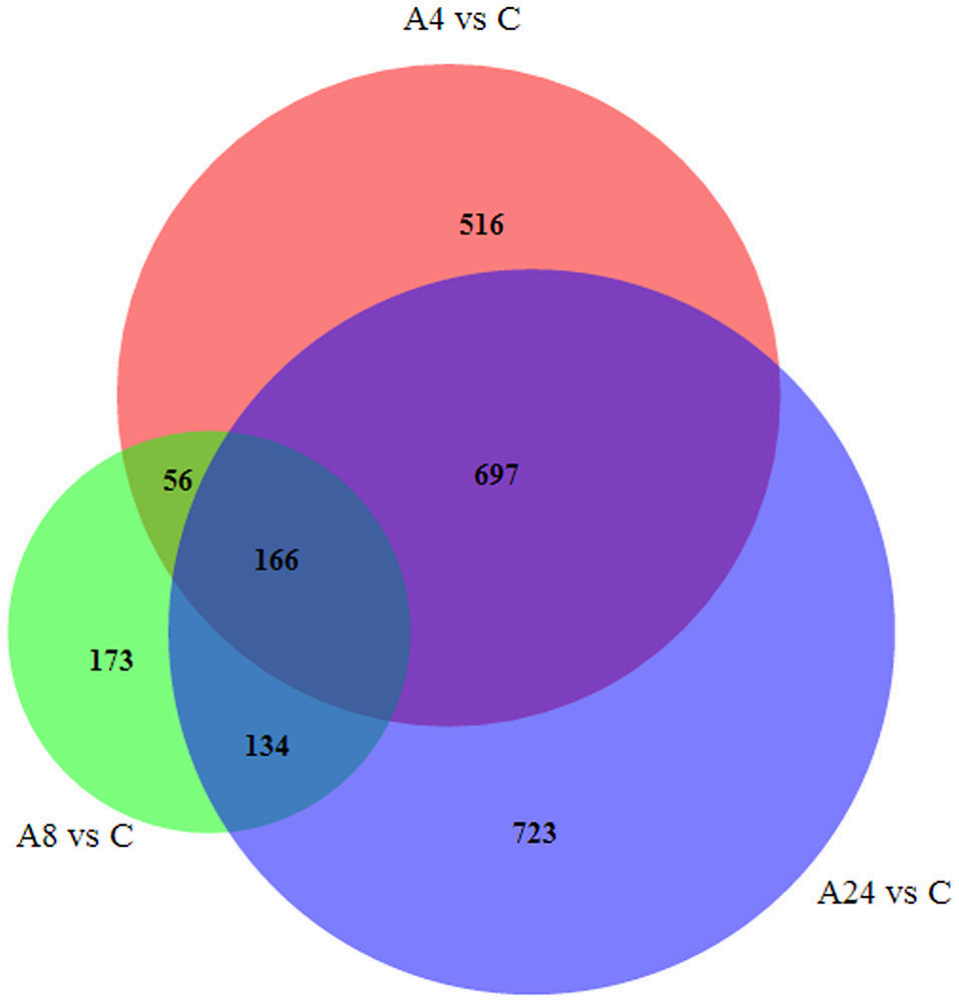
**The number of DEGs expressed at one Al-stress time point and at overlapping time points compared with the control**.

Upon comparison with control group, the unigenes with gene expression fold changes greater than or equal to four and with an FDR value below 10^−4^ were defined as DEGs. Based on these strict criteria, 1435 (1162 up-regulated and 273 down-regulated), 529 (231 up-regulated and 298 down-regulated), and 1702 (1306 up-regulated and 414 down-regulated) DEGs responded to Al stress within the A4, A8, and A24 were detected, respectively (Figures 2A,B), indicated that Al stress caused significant changes in gene expression in alfalfa roots. Particularly, the substantial modulation of gene expression was observed in 4 and 24 h stresses, whereas the number of DGEs in 8 h stress was significantly reduced. In addition, a total of 2464 DEGs were detected after 24 h Al treatment, and 166 were common to all three time points, suggested these genes were continuously significantly modulated during the 24 h Al stress treatment (Figure 3). Furthermore, there were 516, 173, and 723 DEGs specifically modulated in A4, A8, and A24, which representing early, medium, and late responsive DEGs, respectively.

**Figure 4:**
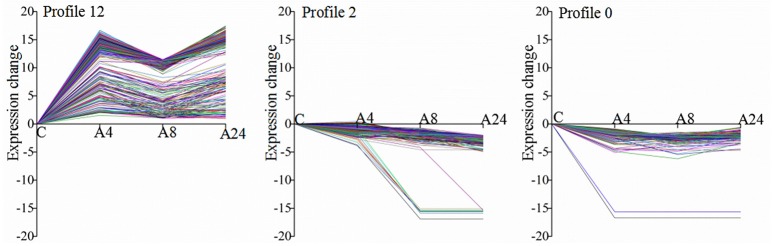
**Differentially expressed gene expression profiles**.

All 2464 DEGs could be clustered into 15 profiles with the STEM software, in which 1618 DEGs were clustered into 3 profiles (*p* ≤ 0.05), including one down-regulated pattern (profile 0 and profile 2) and one up-regulated pattern (profile 12) (Figure 4). Profile 0 and profile 2 contained 133 and 169 DEGs, respectively, while profile 12 contained 1098 DEGs.

### GO functional analysis of the DEGs

A total of 39 GO categories were assigned to the 2464 DEGs that responded to Al treatment (Figure S5). In the biological process category, “metabolic process” (59.7%) was the most dominant group, followed by “cellular process” (56.0%) and “response to stimulus” (27.0%). Regarding the molecular function category, 51.0% of the unigenes were assigned to “binding” followed by “catalytic activity” (48.7%) and “structural molecule activity” (15.1%). In the cellular component category, “cell” and “cell part” (64.3% for both) were the dominant categories, followed by “organelle” (50.9%) and “organelle part” (27.0%).

**Figure 5:**
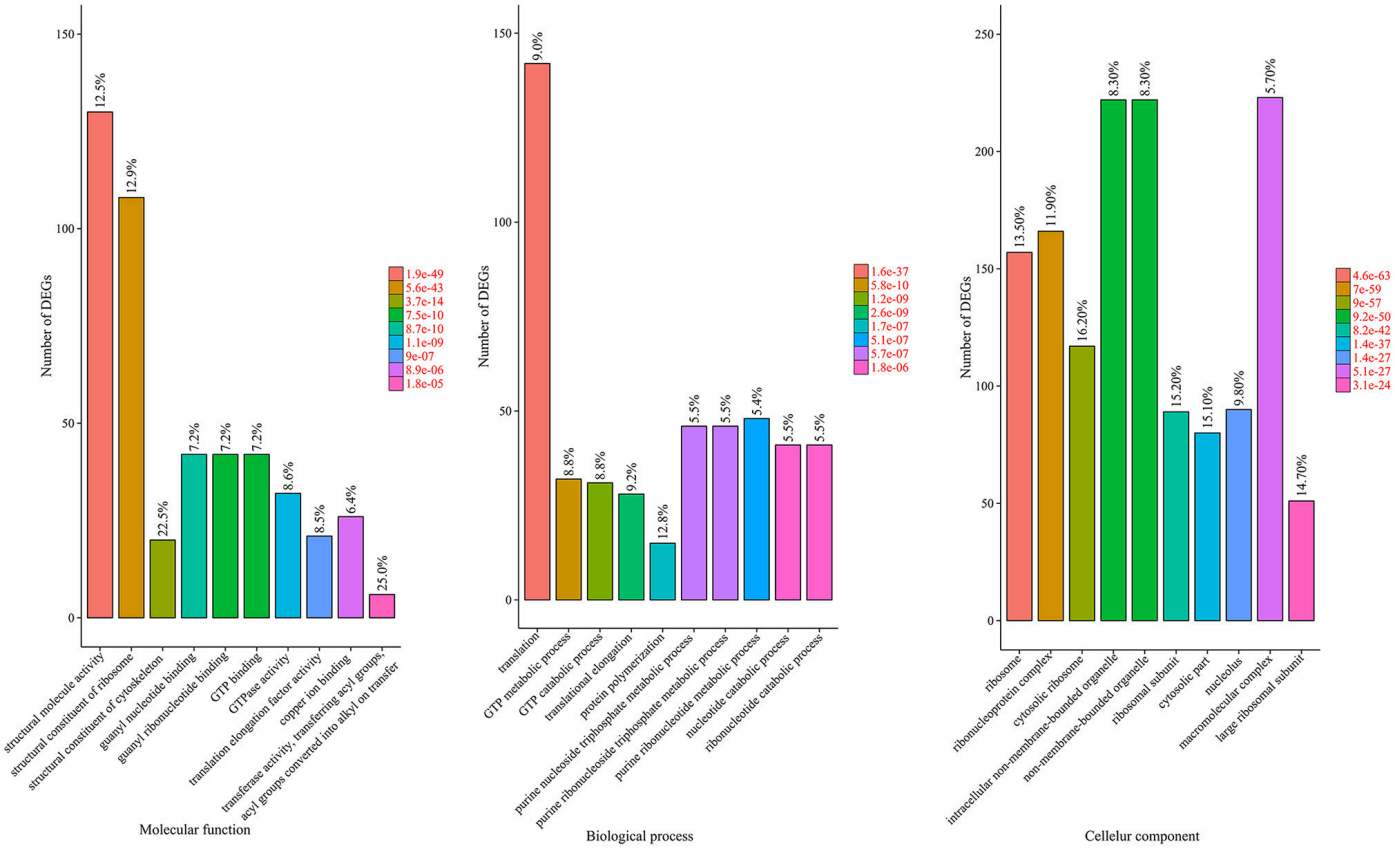
**GO enrichment analysis of the DEGs**. The genes were assigned to three main categories: biological process, molecular function, and cellular component. The names of the GO categories are listed along the x-axis. The degree of GO enrichment is represented by the FDR value and the number of unigenes enriched in each category. The FDR value indicates the corrected *p*-value, ranging from 0 to 1, and an FDR value closer to 0 indicates greater enrichment.

To reveal the significantly enriched GO terms among the DEGs, we used 1006 GO terms annotated from all DEGs as study set and 5288 GO terms annotated from all unigenes as references, and carried out a GO functional-enrichment analysis via the agriGO website with a *p* score cut-off of 0.05. Among the 91 assigned GO terms, 32, 19, and 40 belonged to the “biological process,” “molecular function,” and “cellular component” categories, respectively. The 10 most significantly over-represented GO terms in each category are shown in Figure 5.

### KEGG pathway enrichment analysis of the DEGs

**Figure 6:**
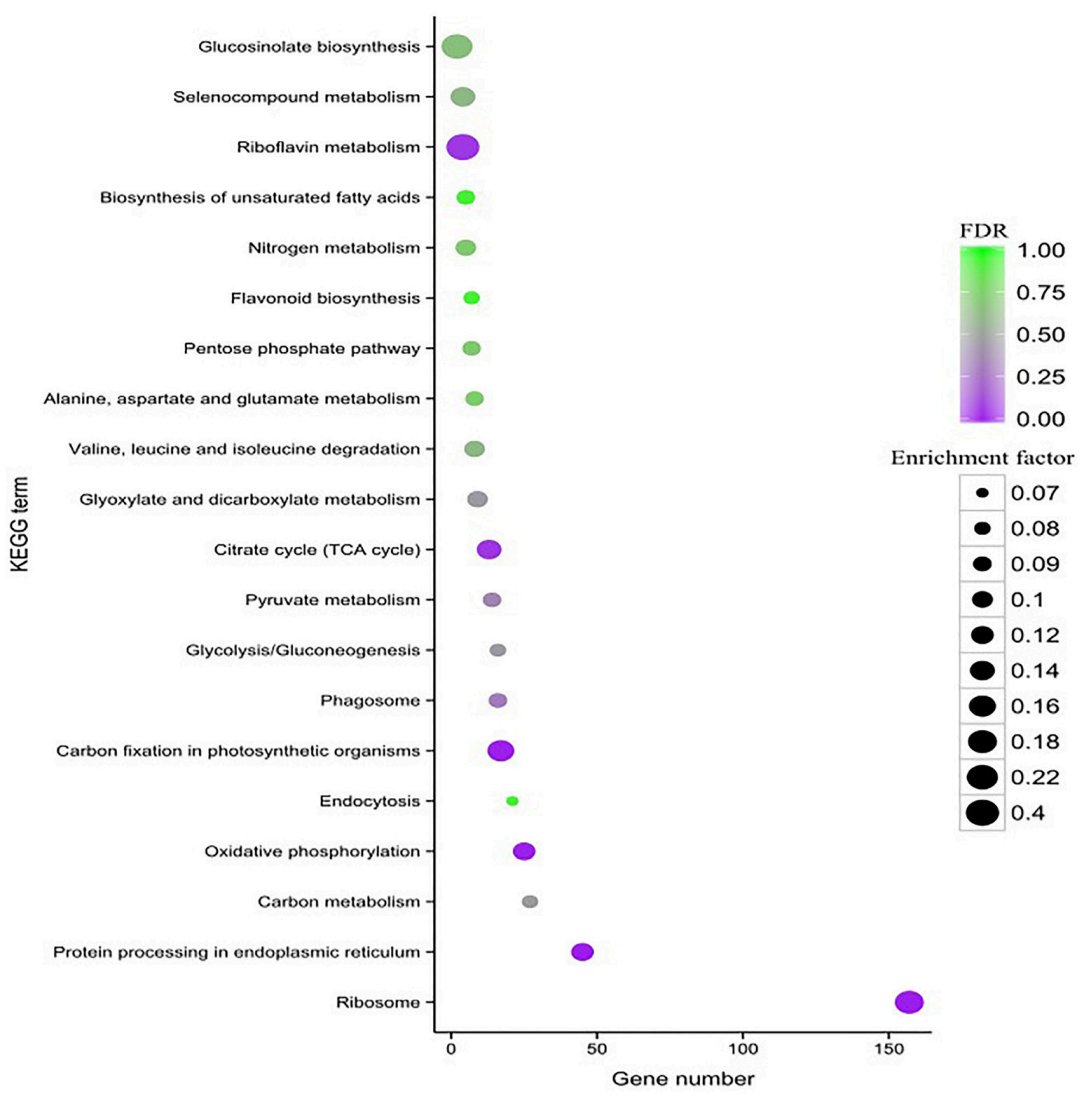
**KEGG pathway enrichment scatter diagram of DEGs**. Only the top 20 most strongly represented pathways are displayed in the diagram. The degree of KEGG pathway enrichment is represented by an enrichment factor, the FDR value, and the number of unigenes enriched in a KEGG pathway. The enrichment factor indicates the ratio of differential expression unigenes enriched in this pathway to the total number of annotated unigenes in this pathway. The names of the KEGG pathways are listed along the y-axis. The FDR value indicates the corrected *p*-value, ranging from 0 to 1, and an FDR value closer to 0 indicates greater enrichment.

To characterize the complex biological behaviors of the transcriptome, all of the DEGs were subjected to a KEGG pathway enrichment analysis. In total, 417 (16.92%) Al stress-responsive DEGs were assigned to 90 different KEGG pathways. The top 20 KEGG pathways with the highest representation of the DEGs are shown in Figure 6. The “ribosome (ko03010),” “protein processing in endoplasmic reticulum (ko04141),” “carbon fixation in photosynthetic organisms (ko00710),” “oxidative phosphorylation (ko00190),” “TCA cycle (ko00020),” and “riboflavin metabolism (ko00740)” categories were significantly enriched.

### Verification of the DEGs

**Figure 7:**
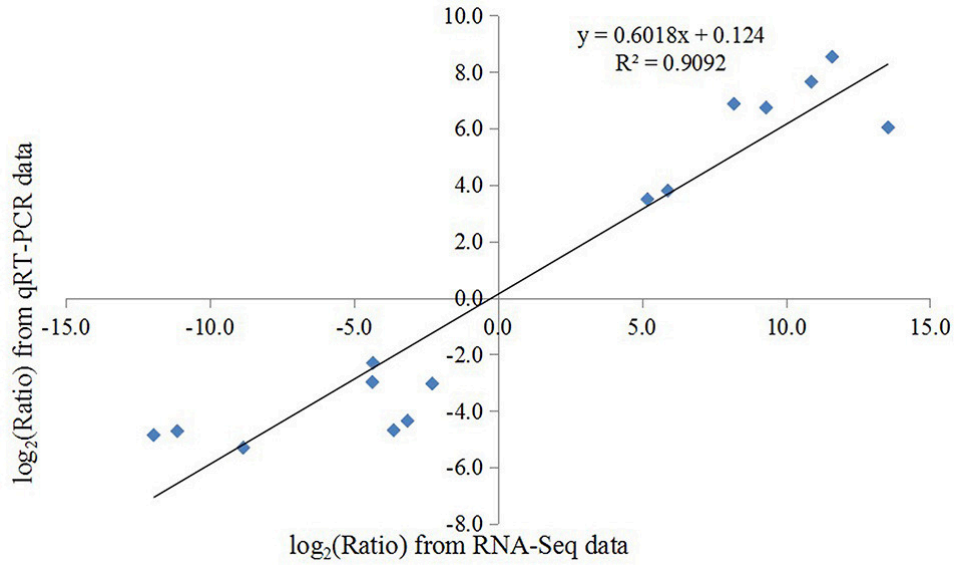
**Validation of the expression changes (log_**2**_-fold) of selected genes from RNA-Seq using qRT-PCR**. The results are plotted for genes that show significant up- or down-regulation in alfalfa roots upon Al stress. The linear trend line and the *R*^2^-value are shown.

To confirm the reliability of our transcriptome data, the expression fold change of 15 candidate DEGs were determined using qRT-PCR and further compared with those of RNA-Seq data. These candidates included 7 up-regulated and 8 down-regulated DEGs and involved in ribosome, TCA cycle, and molecule transport pathways. In our analysis, a positive correlation coefficient (*R*^2^ = 0.9092) was obtained by the linear regression analysis, suggested that the expression of these selected unigenes in our transcriptome data were generally in good agreement with qRT-PCR results (Figure 7).

## Discussion

Plants frequently encounter Al stress in acid soils and have thus evolved a series of responses and adaptive mechanisms to cope with Al stress. Understanding the molecular mechanisms underlying Al stress responses is important for Al-tolerant crop breeding program. Using microarray and newly developed deep sequencing technologies, the transcriptomic responses of many plants to Al stress have been comprehensively documented; however, most of these efforts have focused on model plants, such as *Arabidopsis* (Kumari et al., 2008), soybean (You et al., 2011), maize (Maron et al., 2008; Mattiello et al., 2014), and rice (Arenhart et al., 2014). In the present study, we analyzed the transcript profiles of alfalfa roots in response to Al stress by using the Illumina deep sequencing system and identified a total of 75,903 unigenes in the four sample libraries, which was more than previously reported for alfalfa root transcriptome analyses (Postnikova et al., 2013). Of these unigenes, more than 85% had significant similarity (BLAST, *E* ≤ 10^−5^) to genes in the public databases (Table 2). Using the more stringent criteria of both FDR ≤ 0.0001 and an expression difference greater than four-fold, our results detected 2464 Al stress-related DEGs after treatment with Al for 24 h compared with the control, thus indicating that these genes responded to Al stress in alfalfa. We further classified these Al stress-related DEGs into three groups on the basis of their expression patterns. As shown in Figure 4, most DEGs were up-regulated, a result consistent with those from previous reports in other plants under Al stress (You et al., 2011; Yokosho et al., 2014; Chen et al., 2015). The number of genes down-regulated by Al remained nearly constant over the course of the treatment, whereas the number of up-regulated genes had a dramatically different trend in which many responsive genes were observed at the early (4 h) and/or late (24 h) Al time points compared with those observed at the middle time point (8 h) (Figures 2A,B). These results contrast with those from previous microarray and RNA-Seq analyses of plant response to Al and other types of abiotic stress (Kumari et al., 2008; Xu et al., 2014), suggesting a diverse and complex mechanism by which alfalfa responded to Al stress. Furthermore, the qRT-PCR results showed a significantly positive correlation (*R*^2^ = 0.9092) between the fold-changes of the gene expression ratios obtained by RNA-Seq and those obtained by qRT-PCR (Figure 7), indicating that our RNA-Seq experimental results are valid, and that RNA-Seq data is an accurate method to identify transcripts that are differentially regulated in response to Al. These results will greatly aid in our understanding of the molecular processes associated with Al stress responses and provide further insight for the Al-tolerant alfalfa breeding programs.

Previous studies have shown that some plant species can form sufficiently strong complexes with Al^3+^ to protect the roots from Al stress by releasing organic acids, such as citrate, oxalate, and malate (Kochian et al., 2015). In the present study, the KEGG pathway enrichment analysis of the DEGs indicated that some key genes related to citrate biosynthesis in the TCA cycle were also significantly enriched after Al treatment (Figure 6). These key genes included citrate synthase (CS), phosphoenolpyruvate carboxylase (PEPC), and malate dehydrogenase (MDH). Alterations in the activities of these enzymes may lead to accumulation of citrate in the cytoplasm (Ma et al., 2001). Overexpression of *CS* genes increases citrate efflux in cultured carrot cells (Koyama et al., 1999), *Arabidopsis* (Koyama et al., 2000), canola (Anoop et al., 2003), and tobacco (de la Fuente et al., 1997). When soybean roots are exposed to Al, the activities of mitochondrial MDH and CS have been found to increase in an Al-dose-dependent manner (Xu et al., 2010). In the present study, all of the key genes encoding enzymes involved in citrate biosynthesis mentioned above were identified, and the expression levels of the 4 *PEPC*, 3 *MDH*, and 3 *AC* genes were significantly increased during the Al treatment process (Figure S6A). These results suggested that organic acid production in response to Al occurs in the alfalfa roots.

Currently, two main types of Al resistance mechanisms have been documented that allow plants to cope with Al toxicity: one is Al exclusion mechanism, which prevents Al from entering the root apex (both apoplasm and symplasm) and the Al internal detoxification mechanism, in which Al enters the plant and is detoxified and sequestered (Kochian et al., 2015). Both mechanisms use organic acid anions. In the present transcriptome analysis, although the genes encoding TCA cycle enzymes were up-regulated, the related organic acid transporter genes, which are induced in other plants under Al stress, such as aluminum sensitive malate transporter (ALMT) and NRAMP aluminum transporter 1 (NRAT1), were not found. One gene belonging to the multidrug and toxin extrusion (MATE) family, which has been widely reported to function as citrate transporters in the induction of Al tolerance of many plants (Delhaize et al., 2012), was identified, but its transcript abundance was down-regulated (Figure S6B). These results further support the idea that there is no apparent correlation between the Al-induced expression of organic acid biosynthetic enzymes and increased exudation of organic acids (Chandran et al., 2008) and that the biosynthesis rather than exudation of organic acids is more critical for Al response in alfalfa roots. However, one gene and 8 genes that showing similarity to the aluminum-sensitive 1 (ALS1) and major facilitator superfamily (MFS) protein genes, respectively, were identified (Figure S6B). Previous studies have shown that these genes are involved in internal Al detoxification mechanisms by sequestering the Al-organic acid anion complexes inside the vacuoles of root cells (Huang et al., 2012; Yokosho et al., 2014). The up-regulation of these two types of genes in our RNA-Seq data suggested their involvement in the internal Al detoxification mechanism, although further functional analysis is required. In addition to the organic acid transporters, transporters for other small molecules/ions were also found to be up-regulated in alfalfa roots (Figure S6B), such as sugar transporters, sulfate transporters, vacuolar iron transporters, zinc transporters, and nitrate transporters, thus indicating that the uptake and translocation of other nutrients is affected by Al stress. Similar results have also been observed in buckwheat (Yokosho et al., 2014) and hydrangea (Chen et al., 2015), and additional investigations are necessary for further understanding this mechanism of Al responses in alfalfa.

Ribosomes are essential ribonucleoprotein complexes that are engaged in protein synthesis and thus are indispensable for metabolism, cell division, and growth (Wang J. Y. et al., 2013). In addition to their housekeeping functions, increasing evidence has suggested that ribosomal proteins also play more regulatory roles in leaf development, auxin responsiveness, wounding, and biotic/ abiotic stress responses (Liu et al., 2016). It has been reported that ribosomal protein genes can be differentially regulated by various environmental conditions. For example, up-regulation of several ribosomal proteins genes has been observed in maize exposed to UV-B light (Casati and Walbot, 2003), whereas the DEGs that respond to Al stress in the maize root are down-regulated (Maron et al., 2008). In *Anthoxanthum*, both up-regulated and down-regulated DEGs were identified in tolerant and sensitive genotypes when exposed to Al treatment (Gould et al., 2015). Microarray analyses have revealed that the transcript abundance for 5 ribosomal genes is increased after 6 h Al treatment, whereas after 48 h exposure, the transcripts for 5 ribosomal genes increase and the transcripts for 3 ribosomal genes decrease in abundance, thus indicating that there may be increased demand for specific ribosomal components during Al exposure (Kumari et al., 2008). In the present study, the KEGG pathway with the largest number of significantly enriched DEGs was “ribosome.” In contrast to previous studies in which fewer Al-response DEGs were detected, many more (157) DEGs were identified in our study, and most are components of large or small ribosomal subunits (Figure S6C). Among these DEGs, the transcript abundances for 93 DEGs were up-regulated during the 24 h treatment, and the remaining DEGs were down-regulated, which suggested a high biological importance for ribosomal genes in response to Al stress in alfalfa.

Heat Shock Proteins (HSPs) are important in stabilizing, folding, and degrading damaged proteins. In addition, numerous studies have determined that HSPs also function as molecular chaperones and protect plants from damage under stress conditions, such as Al- (Kumari et al., 2008; Duressa et al., 2011), NaCl- (Jiang and Deyholos, 2006), and heat temperature-induced stress (Sung et al., 2001). In the present study, there were 28 transcripts encoding HSPs (14 HSP70s, 7 HSP20s, 5 HSPs, and 2 HSP80s) that were enriched in the “protein processing in endoplasm reticulum” KEGG pathway (Figure S6D), which was the second most significantly enriched term. HSP70s is one of the most important members of the HSP protein family. The major role of HSP70 is to prevent protein aggregation and assist in protein refolding, import, translocation, signal transduction, and transcriptional activation (Wang Y. et al., 2013). In this transcriptome analysis, more than 53.8% of the HSPs were HSP70s, and 8 of these genes were up-regulated, whereas 6 were down-regulated, thus indicating that HSP70 may play important roles in Al stress responses in alfalfa.

Plant hormones are signaling molecules that regulate a wide range of metabolic and development processes at extremely low concentrations. Ethylene mediates root growth inhibition, whereas increased auxin biosynthesis in roots further facilitates ethylene-dependent growth inhibition (Swarup et al., 2007). Al has also been shown to affect root growth by modifying the levels of auxin (Ponce et al., 2005) and ethylene (Sun et al., 2007). It is well known that auxin response factors (ARFs) are a family of transcription factors that specifically bind to auxin-response elements of primary/early auxin response genes, whereas auxin-responsive proteins (AUX/IAA) repress the activity of ARFs, and thus this interaction plays a pivotal and concerted role in regulating the auxin response pathway (Dharmasiri and Estelle, 2004). Small auxin up RNA (SAUR) genes can be readily induced by exogenous auxins and function as negative regulators of auxin synthesis and transport (Kant et al., 2009). Ethylene responsive factors (ERFs) are plant-specific transcription factors belonging to the AP2/ERF family and have been reported to bind to the GCC-box found in the promoter of ethylene-inducible genes, thus acting as transcriptional activators of the ethylene response cascade and protecting cells from damage caused by metal stresses in plants (Makhloufi et al., 2014). In this transcriptome analysis, genes encoding enzymes involved in auxin homeostasis and response pathways, such as *SAUR* (CL3536.Contig2_All) and *AUX/IAA* (Unigene9889_All) were down-regulated, whereas the *ARF* (Unigene30886_All) was up-regulated through the Al treatment. In addition, the transcript abundances of most *ERF* genes were also increased (Figure S6E). These results suggested that both the inhibition of auxin production and the downstream signal transduction of these two hormones may be adopted in alfalfa in response to the root growth inhibition caused by Al stress.

## Author contributions

ZL and WL conceived and designed the study. WL, LY, ZZ, LM, and YL performed the experiments. WL, CX and ZL analyzed the data. WL, CX and YW wrote the paper. All of the authors read and approved the final manuscript.

## Availability of supporting data

The raw sequence data supporting the results of this article are available in the Short Read Archive (SRA) (accession number SRS654614), http://www.ncbi.nlm.nih.gov/sra.

### Conflict of interest statement

The authors declare that the research was conducted in the absence of any commercial or financial relationships that could be construed as a potential conflict of interest.

## Supplementary material

Figure S1 **The quality of the assembled transcripts**.

Figure S2 **Venn diagram of the unigenes in the four libraries**.

Figure S3 **COG function classifications of the assembled transcripts**.

Figure S4 **KEGG annotation of the assembled transcripts**.

Figure S5 **GO classification of the DEGs**. The genes were assigned to three main categories: biological process, molecular function and cellular component.

Figure S6 **Heat map diagram of expression levels of DEGs specifically enriched in KEGG pathways. (A)** DEGs involved in the TCA cycle. **(B)** DEGs involved in transport. **(C)** DEGs involved in ribosome. **(D)** DEGs involved in protein processing in the endoplasm reticulum. **(E)** DEGs involved in plant hormone signal transduction.

Table S1 **Primers used for qRT-PCR analysis**.

Table S2 **GO classification of the DEGs**. The genes were assigned to three main categories: biological process, cellular process and molecular function.
