# Supplementary figures and images for: Transcriptome Analyses Reveal Candidate Genes Potentially Involved in Al Stress Response in Alfalfa
Source: Front Plant Sci. 2017 Feb 2;8:26. doi: 10.3389/fpls.2017.00026 (PMC5290290; doi:10.3389/fpls.2017.00026)

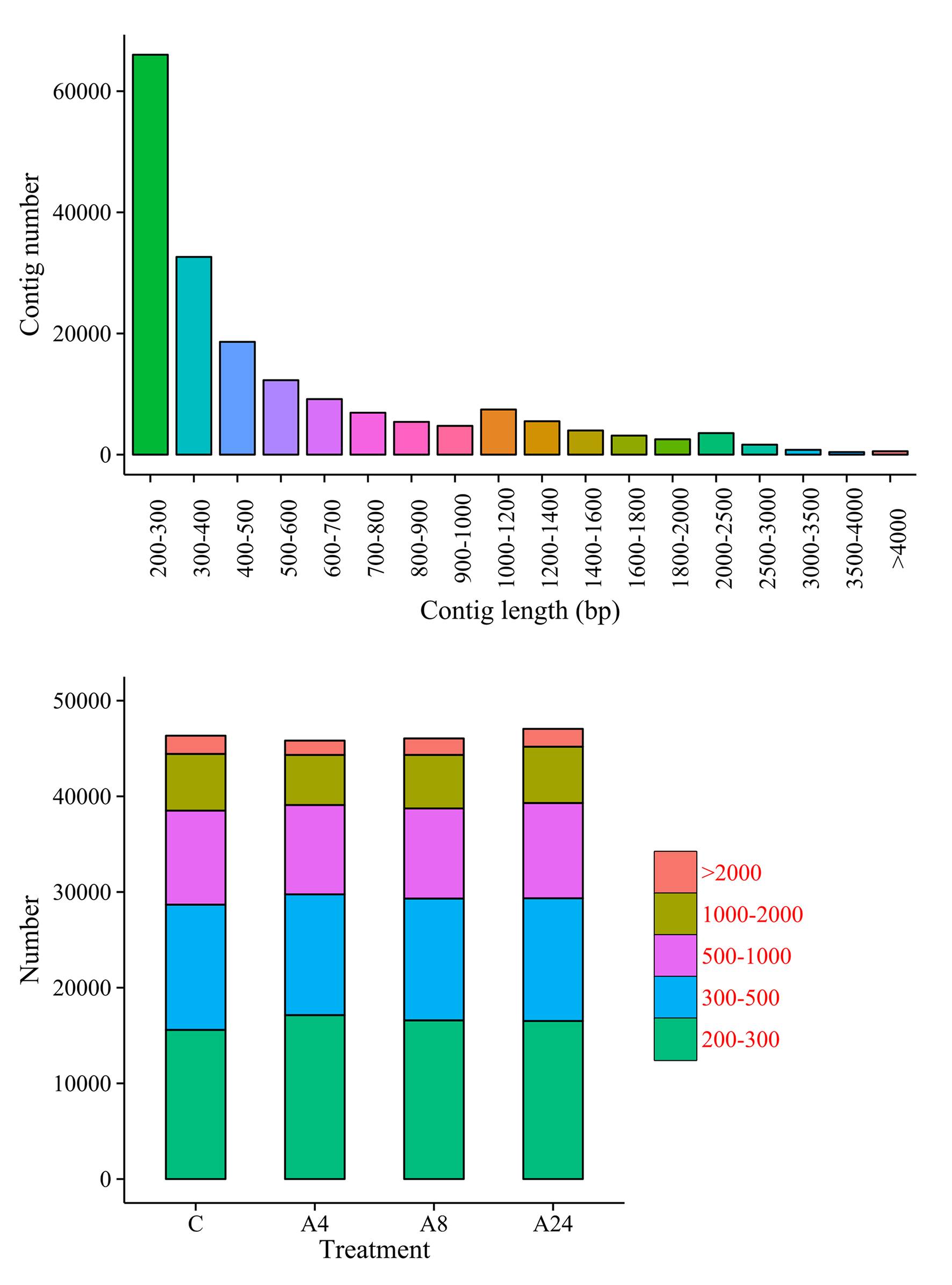

Supplement: Figure S1 — The quality of the assembled transcripts. [file Image1.TIF]

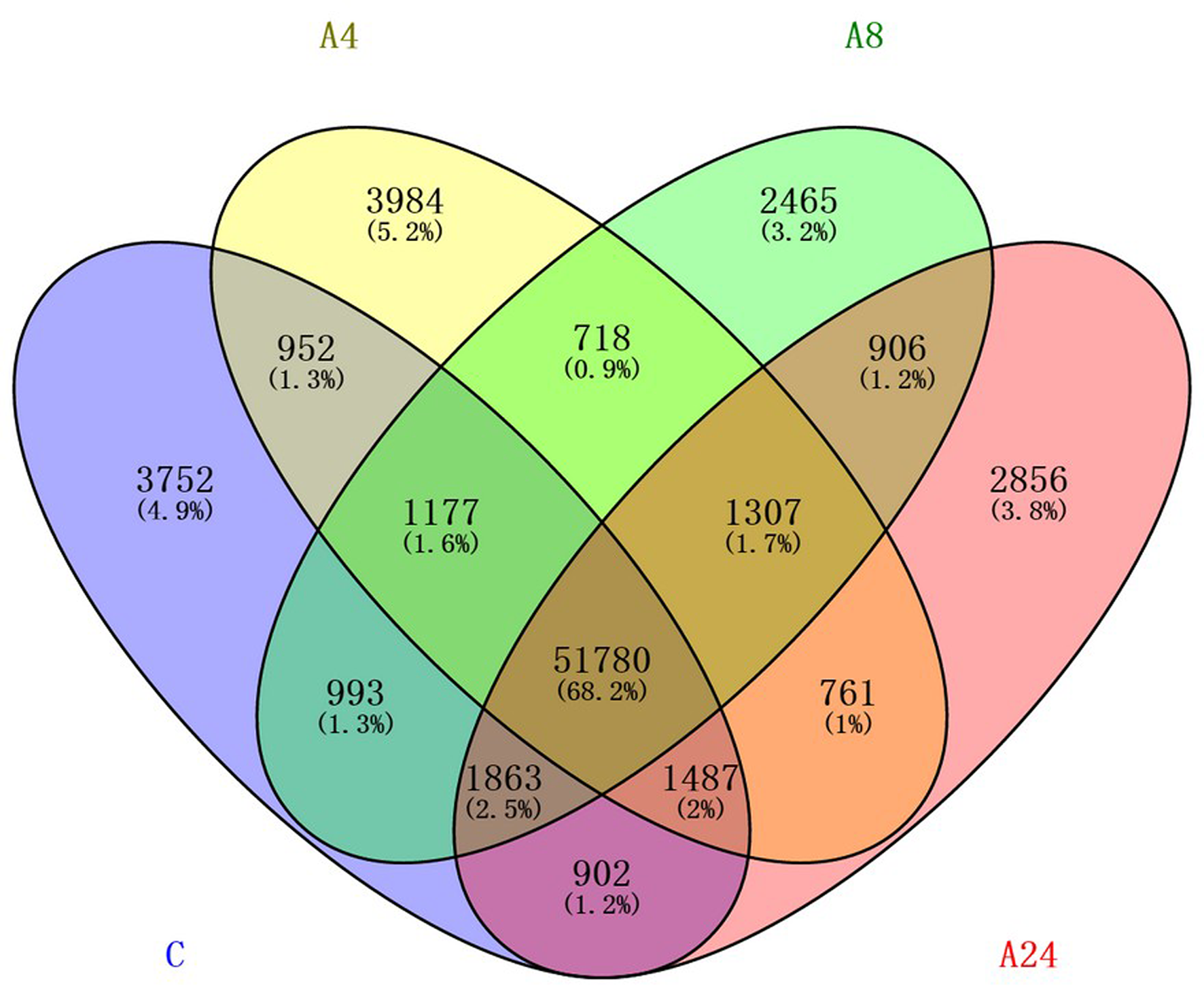

Supplement: Figure S2 — Venn diagram of the unigenes in the four libraries. [file Image2.TIF]

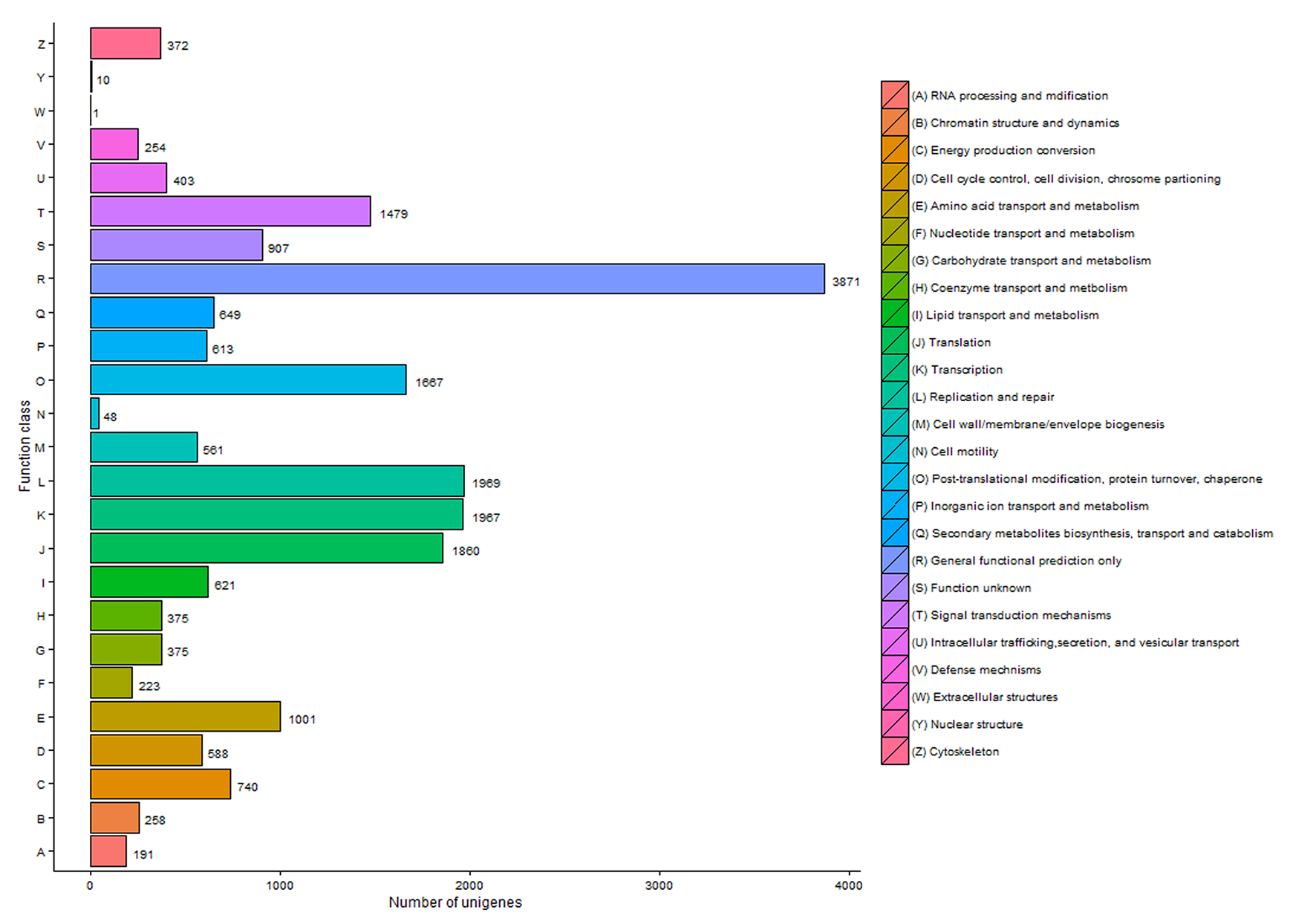

Supplement: Figure S3 — COG function classifications of the assembled transcripts. [file Image3.TIF]

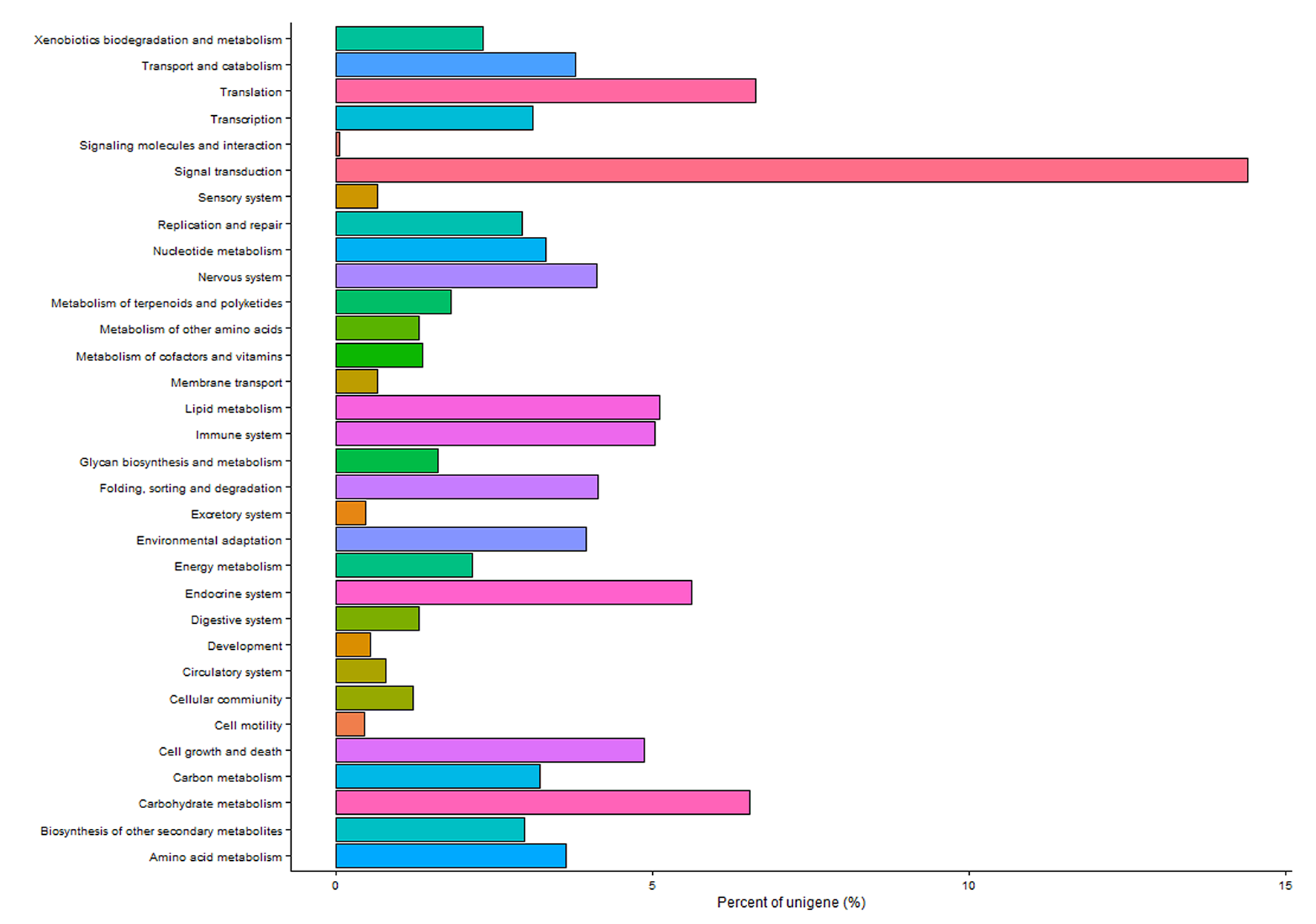

Supplement: Figure S4 — KEGG annotation of the assembled transcripts. [file Image4.TIF]

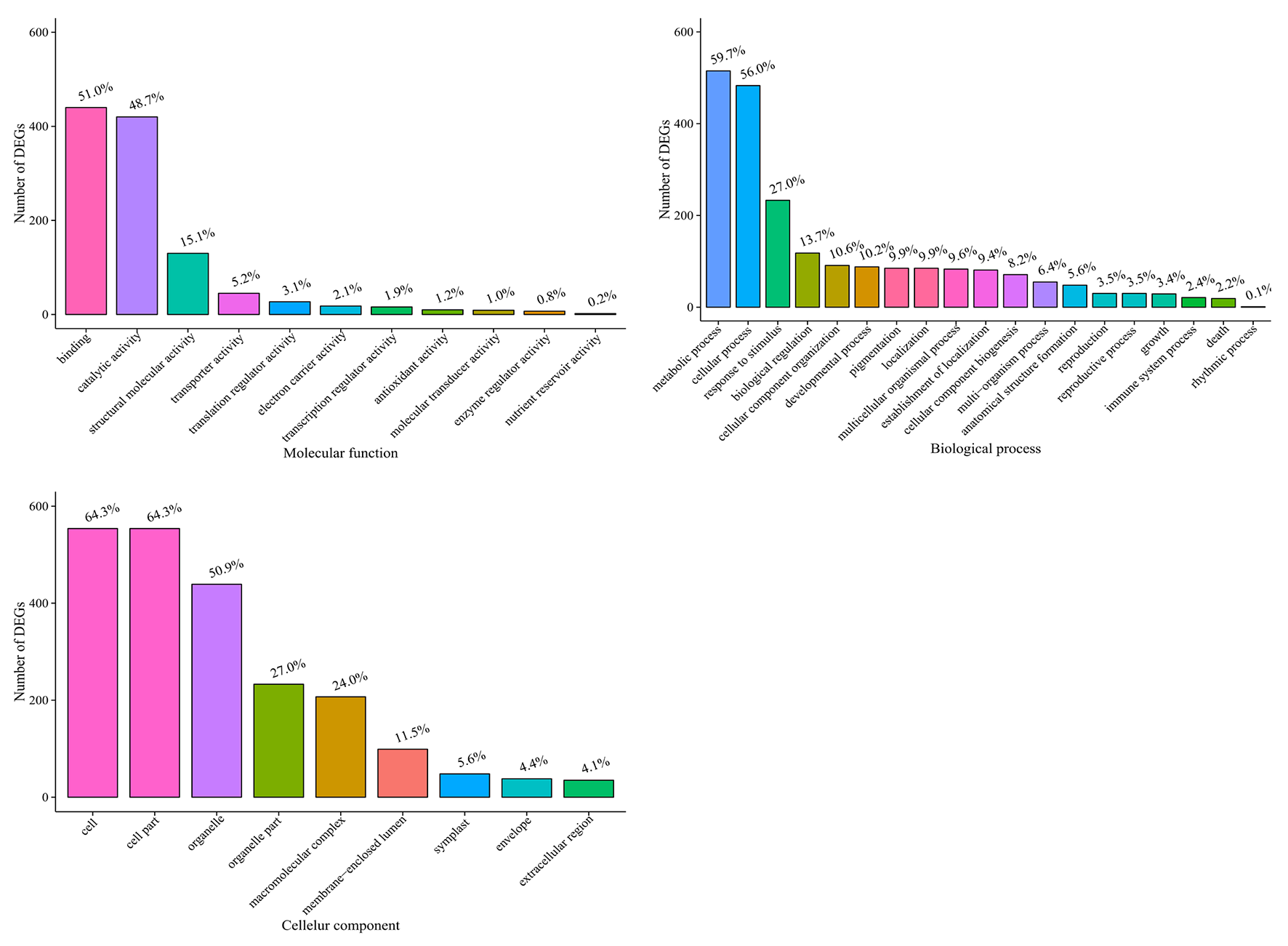

Supplement: Figure S5 — GO classification of the DEGs. The genes were assigned to three main categories: biological process, molecular function and cellular component. [file Image5.TIF]
