# Supplementary material for: Transcriptome Analyses Reveal Candidate Genes Potentially Involved in Al Stress Response in Alfalfa
Source: Front Plant Sci. 2017 Feb 2;8:26. doi: 10.3389/fpls.2017.00026 (PMC5290290; doi:10.3389/fpls.2017.00026)
Supplement: Figure S6 — Heat map diagram of expression levels of DEGs specifically enriched in KEGG pathways. (A) DEGs involved in the TCA cycle. (B) DEGs involved in transport. (C) DEGs involved in ribosome. (D) DEGs involved in protein processing in the endoplasm reticulum. (E) DEGs involved in plant hormone signal transduction. [file Image6.PDF]

(A)

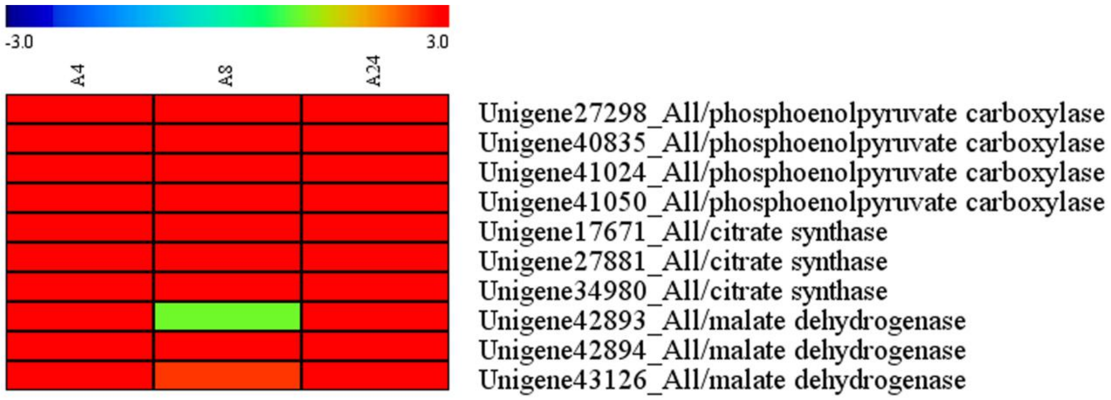
-3.0
3.0
A4
A8
A24
Unigene27298_All/phosphoenolpyruvate carboxylase
Unigene40835_All/phosphoenolpyruvate carboxylase
Unigene41024_All/phosphoenolpyruvate carboxylase
Unigene41050_All/phosphoenolpyruvate carboxylase
Unigene17671_All/citrate synthase
Unigene27881_All/citrate synthase
Unigene34980_All/citrate synthase
Unigene42893_All/malate dehydrogenase
Unigene42894_All/malate dehydrogenase
Unigene43126_All/malate dehydrogenase

(B)

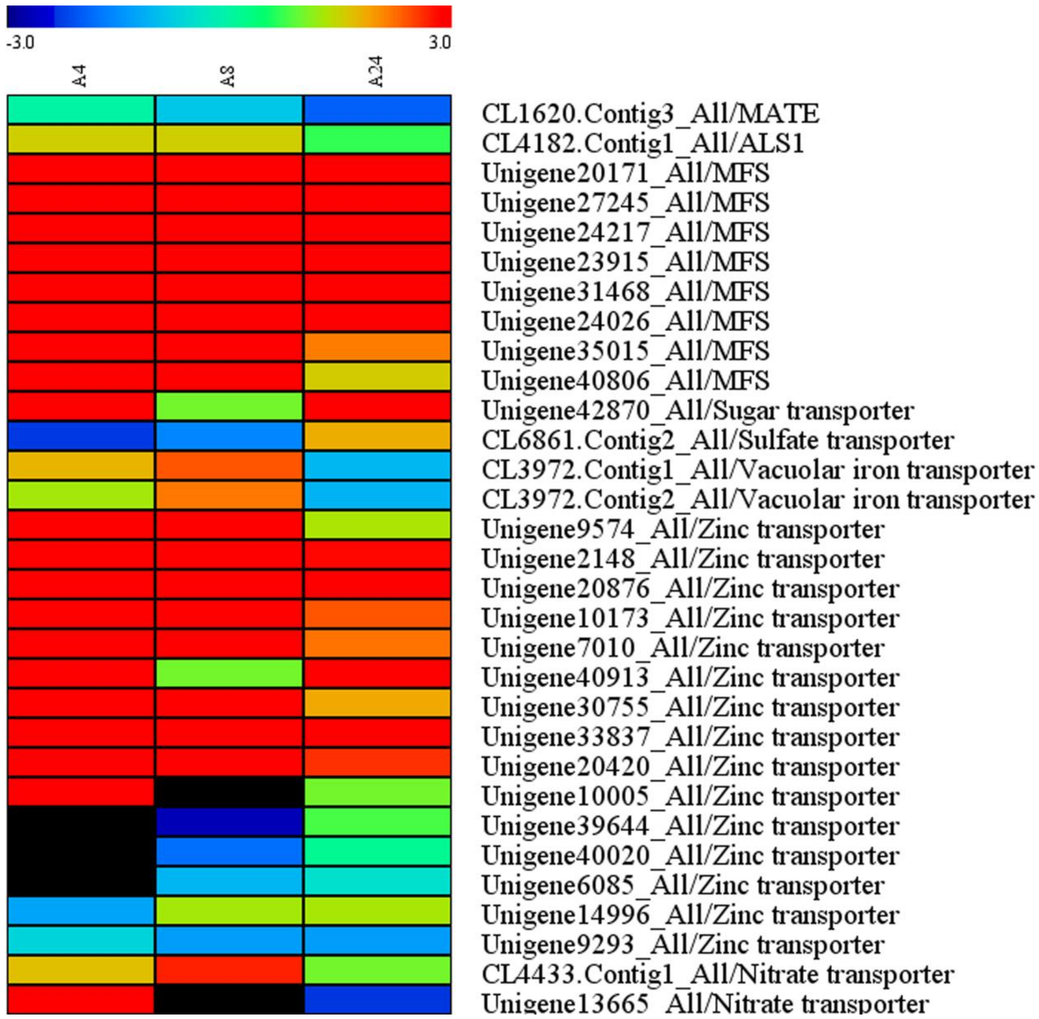
-3.0
3.0
A4
A8
A24
CL1620.Contig3_All/MATE
CL4182.Contig1_All/ALS1
Unigene20171_All/MFS
Unigene27245_All/MFS
Unigene24217_All/MFS
Unigene23915_All/MFS
Unigene31468_All/MFS
Unigene24026_All/MFS
Unigene35015_All/MFS
Unigene40806_All/MFS
Unigene42870_All/Sugar transporter
CL6861.Contig2_All/Sulfate transporter
CL3972.Contig1_All/Vacuolar iron transporter
CL3972.Contig2_All/Vacuolar iron transporter
Unigene9574_All/Zinc transporter
Unigene2148_All/Zinc transporter
Unigene20876_All/Zinc transporter
Unigene10173_All/Zinc transporter
Unigene7010_All/Zinc transporter
Unigene40913_All/Zinc transporter
Unigene30755_All/Zinc transporter
Unigene33837_All/Zinc transporter
Unigene20420_All/Zinc transporter
Unigene10005_All/Zinc transporter
Unigene39644_All/Zinc transporter
Unigene40020_All/Zinc transporter
Unigene6085_All/Zinc transporter
Unigene14996_All/Zinc transporter
Unigene9293_All/Zinc transporter
CL4433.Contig1_All/Nitrate transporter
Unigene13665_All/Nitrate transporter

(C)

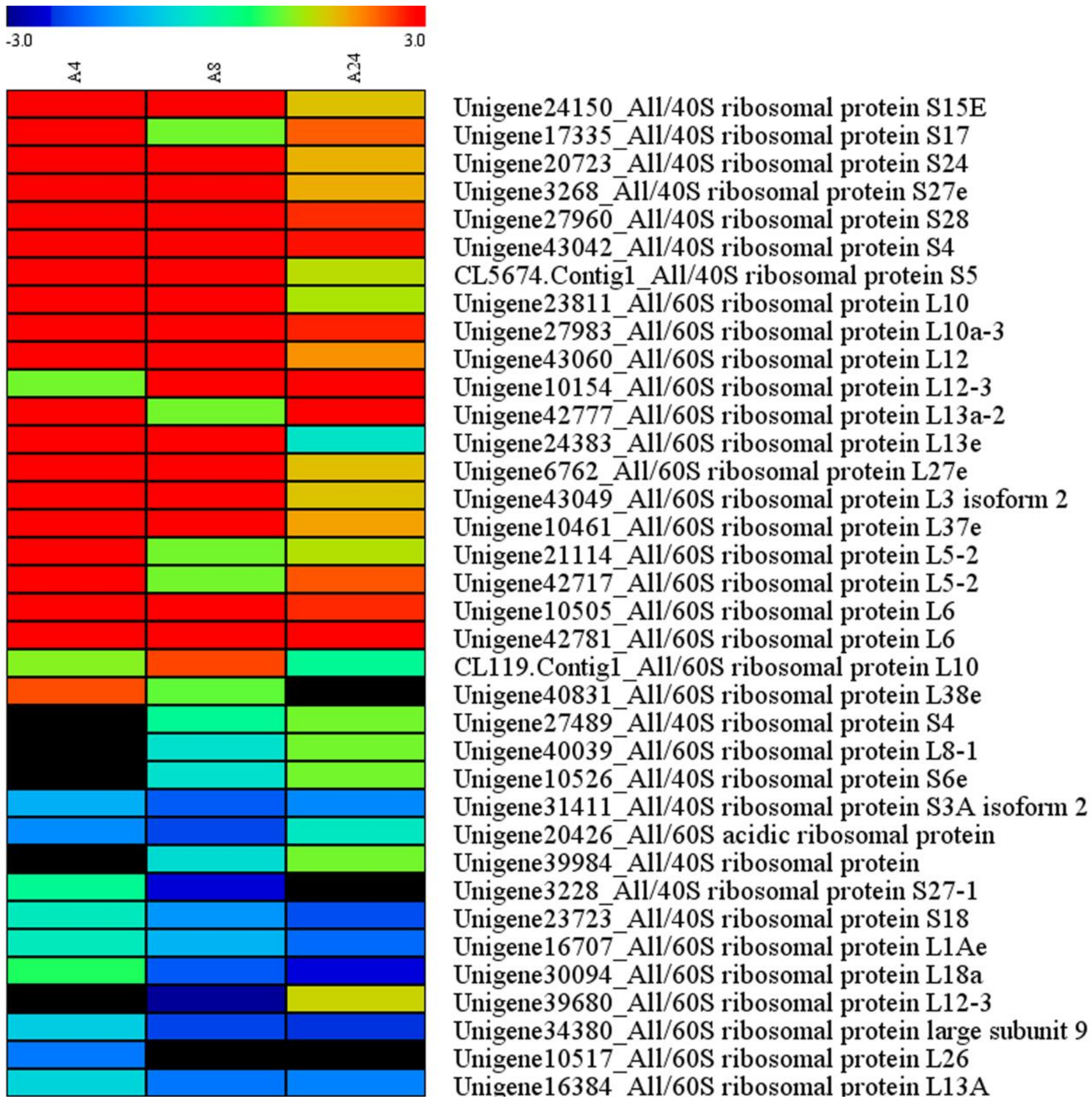
-3.0
3.0
A4
A8
A24
Unigene24150_All/40S ribosomal protein S15E
Unigene17335_All/40S ribosomal protein S17
Unigene20723_All/40S ribosomal protein S24
Unigene3268_All/40S ribosomal protein S27e
Unigene27960_All/40S ribosomal protein S28
Unigene43042_All/40S ribosomal protein S4
CL5674.Contig1_All/40S ribosomal protein S5
Unigene23811_All/60S ribosomal protein L10
Unigene27983_All/60S ribosomal protein L10a-3
Unigene43060_All/60S ribosomal protein L12
Unigene10154_All/60S ribosomal protein L12-3
Unigene42777_All/60S ribosomal protein L13a-2
Unigene24383_All/60S ribosomal protein L13e
Unigene6762_All/60S ribosomal protein L27e
Unigene43049_All/60S ribosomal protein L3 isoform 2
Unigene10461_All/60S ribosomal protein L37e
Unigene21114_All/60S ribosomal protein L5-2
Unigene42717_All/60S ribosomal protein L5-2
Unigene10505_All/60S ribosomal protein L6
Unigene42781_All/60S ribosomal protein L6
CL119.Contig1_All/60S ribosomal protein L10
Unigene40831_All/60S ribosomal protein L38e
Unigene27489_All/40S ribosomal protein S4
Unigene40039_All/60S ribosomal protein L8-1
Unigene10526_All/40S ribosomal protein S6e
Unigene31411_All/40S ribosomal protein S3A isoform 2
Unigene20426_All/60S acidic ribosomal protein
Unigene39984_All/40S ribosomal protein
Unigene3228_All/40S ribosomal protein S27-1
Unigene23723_All/40S ribosomal protein S18
Unigene16707_All/60S ribosomal protein L1Ae
Unigene30094_All/60S ribosomal protein L18a
Unigene39680_All/60S ribosomal protein L12-3
Unigene34380_All/60S ribosomal protein large subunit 9
Unigene10517_All/60S ribosomal protein L26
Unigene16384_All/60S ribosomal protein L13A

(D)

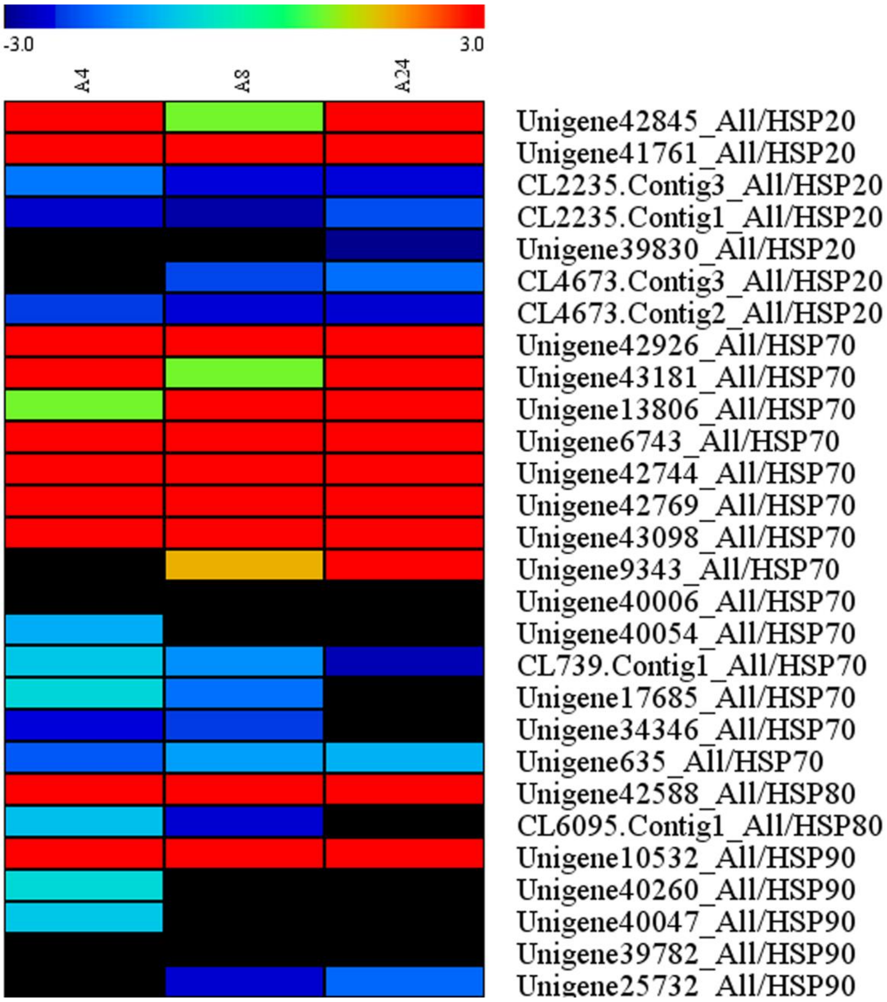
-3.0
3.0
A4
A8
A24
Unigene42845_All/HSP20
Unigene41761_All/HSP20
CL2235.Contig3_All/HSP20
CL2235.Contig1_All/HSP20
Unigene39830_All/HSP20
CL4673.Contig3_All/HSP20
CL4673.Contig2_All/HSP20
Unigene42926_All/HSP70
Unigene43181_All/HSP70
Unigene13806_All/HSP70
Unigene6743_All/HSP70
Unigene42744_All/HSP70
Unigene42769_All/HSP70
Unigene43098_All/HSP70
Unigene9343_All/HSP70
Unigene40006_All/HSP70
Unigene40054_All/HSP70
CL739.Contig1_All/HSP70
Unigene17685_All/HSP70
Unigene34346_All/HSP70
Unigene635_All/HSP70
Unigene42588_All/HSP80
CL6095.Contig1_All/HSP80
Unigene10532_All/HSP90
Unigene40260_All/HSP90
Unigene40047_All/HSP90
Unigene39782_All/HSP90
Unigene25732_All/HSP90

(E)

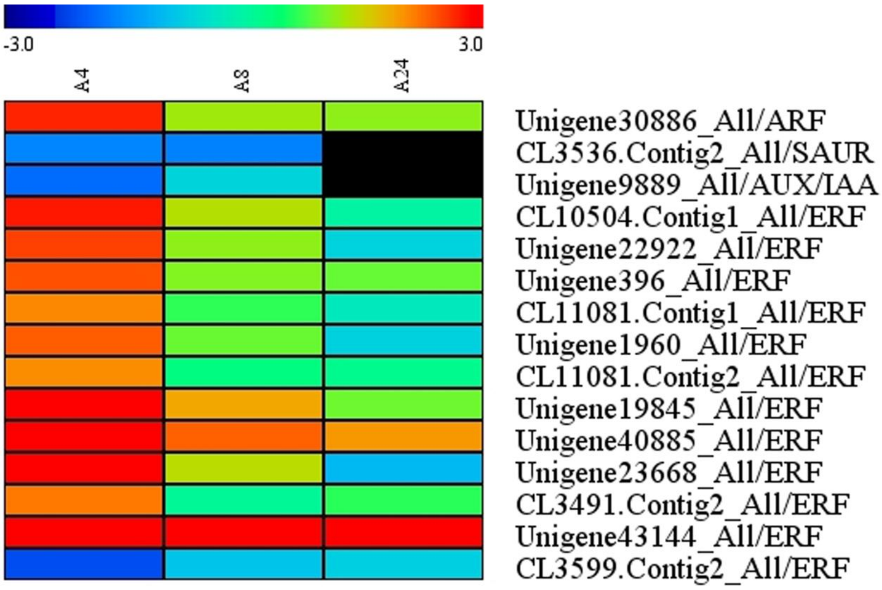
-3.0
3.0
A4
A8
A24
Unigene30886_All/ARF
CL3536.Contig2_All/SAUR
Unigene9889_All/AUX/IAA
CL10504.Contig1_All/ERF
Unigene22922_All/ERF
Unigene396_All/ERF
CL11081.Contig1_All/ERF
Unigene1960_All/ERF
CL11081.Contig2_All/ERF
Unigene19845_All/ERF
Unigene40885_All/ERF
Unigene23668_All/ERF
CL3491.Contig2_All/ERF
Unigene43144_All/ERF
CL3599.Contig2_All/ERF
